# Functional neuronal network activity differs with cognitive dysfunction in childhood-onset systemic lupus erythematosus

**DOI:** 10.1186/ar4197

**Published:** 2013-03-07

**Authors:** Mark W DiFrancesco, Darren R Gitelman, Marisa S Klein-Gitelman, Anna Carmela P Sagcal-Gironella, Frank Zelko, Dean Beebe, Todd Parrish, Jessica Hummel, Jun Ying, Hermine I Brunner

**Affiliations:** 1 Pediatric Neuroimaging Research Consortium, Cincinnati Children's Hospital Medical Center (CCHMC), 3333 Burnet Ave., Cincinnati, OH 45229, USA; 2 Department of Neurology, Feinberg School of Medicine, Northwestern University, 420 East Superior St., Chicago, IL 60611, USA; 3 Department of Radiology, Feinberg School of Medicine, Northwestern University, 420 East Superior St., Chicago, IL 60611, USA; 4 Division of Rheumatology, Ann & Robert Lurie Children's Hospital, Feinberg School of Medicine, Northwestern University, 420 East Superior St., Chicago, IL 60611, USA; 5 Division of Rheumatology, Cincinnati Children's Hospital Medical Center (CCHMC), 3333 Burnet Ave., Cincinnati, OH 45229, USA; 6 Department of Child and Adolescent Psychiatry, Ann & Robert Lurie Children's Hospital, Feinberg School of Medicine, Northwestern University, 420 East Superior St., Chicago, IL 60611, USA; 7 Division of Behavioral Medicine and Child Psychology, Cincinnati Children's Hospital Medical Center (CCHMC), 3333 Burnet Ave., Cincinnati, OH 45229, USA; 8 Department of Public Health, University of Cincinnati, 2600 Clifton Ave., Cincinnati, OH 45221, USA

## Abstract

**Introduction:**

Neuropsychiatric manifestations are common in childhood-onset systemic lupus erythematosus (cSLE) and often include neurocognitive dysfunction (NCD). Functional magnetic resonance imaging (fMRI) can measure brain activation during tasks that invoke domains of cognitive function impaired by cSLE. This study investigates specific changes in brain function attributable to NCD in cSLE that have potential to serve as imaging biomarkers.

**Methods:**

Formal neuropsychological testing was done to measure cognitive ability and to identify NCD. Participants performed fMRI tasks probing three cognitive domains impacted by cSLE: visuoconstructional ability (VCA), working memory, and attention. Imaging data, collected on 3-Tesla scanners, included a high-resolution T1-weighted anatomic reference image followed by a T2*-weighted whole-brain echo planar image series for each fMRI task. Brain activation using blood oxygenation level-dependent contrast was compared between cSLE patients with NCD (NCD-group, *n *= 7) vs. without NCD (noNCD-group, *n *= 14) using voxel-wise and region of interest-based analyses. The relationship of brain activation during fMRI tasks and performance in formal neuropsychological testing was assessed.

**Results:**

Greater brain activation was observed in the noNCD-group vs. NCD-group during VCA and working memory fMRI tasks. Conversely, compared to the noNCD-group, the NCD-group showed more brain activation during the attention fMRI task. In region of interest analysis, brain activity during VCA and working memory fMRI tasks was positively associated with the participants' neuropsychological test performance. In contrast, brain activation during the attention fMRI task was negatively correlated with neuropsychological test performance. While the NCD group performed worse than the noNCD group during VCA and working memory tasks, the attention task was performed equally well by both groups.

**Conclusions:**

NCD in patients with cSLE is characterized by differential activation of functional neuronal networks during fMRI tasks probing working memory, VCA, and attention. Results suggest a compensatory mechanism allows maintenance of attentional performance under NCD. This mechanism appears to break down for the VCA and working memory challenges presented in this study. The observation that neuronal network activation is related to the formal neuropsychological testing performance makes fMRI a candidate imaging biomarker for cSLE-associated NCD.

## Introduction

Studies suggest that neuropsychiatric systemic lupus erythematosus (NPSLE) is present in as many as 80% of adults with SLE [1-3] and may be even more common in childhood-onset SLE (cSLE) [3,4]. The etiology of NPSLE in both children and adults remains the focus of intense research. Neurocognitive dysfunction (NCD) is one of the many manifestations of NPSLE and is encountered in up to 59% of all children with cSLE, often impairing attention, visuoconstructional ability (VCA), and working memory [3], although conventional structural brain imaging often fails to identify matching pathology.

Brain function can now be mapped using blood oxygenation level-dependent (BOLD) functional magnetic resonance imaging (fMRI) that utilizes deoxyhemoglobin as an endogenous contrast agent to identify areas of altered perfusion. The coupling of neuronal activity to hemodynamics allows the identification of neuronal networks whose activity changes during the performance of cognitive tasks [5]. Our own pilot study suggested differences in neuronal network activation in patients with cSLE when compared to healthy controls [6]. However, the association between neuronal network changes and the degree and types of cognitive impairment encountered in cSLE has not been well examined. Thus, the objective of this study was to use fMRI to characterize differences in neuronal network activation that distinguish patients with cSLE-associated NCD from cSLE patients with normal cognition.

## Methods

For this cross-sectional study, participants were recruited from two study sites (Cincinnati and Chicago). The study was approved by the institutional review boards of Cincinnati Children's Hospital Medical Center, Ann and Robert H. Lurie Children's Hospital of Chicago, and Northwestern University. Assent and written parental consent were obtained prior to any study procedure.

### Patients

All participants fulfilled the American College of Rheumatology classification criteria for SLE prior to age 16 years [7] and were between the ages of 9 and 18 years at the time of the study. Excluded from participation were cSLE patients with a history, prior to the diagnosis of cSLE, of comorbid conditions affecting neurocognitive function, the presence of known structural brain abnormalities, neuropathies, movement disorders, or seizures.

Sociodemographic status was assessed for each participant and medical histories were reviewed for information relevant to cSLE. Disease activity and damage were measured by the SLE Disease Activity Index and the Systemic Lupus International Collaborating Clinics/American College of Rheumatology Damage Index [8], respectively.

### Measurement of cognitive ability and definition of NCD

Formal neuropsychological testing was performed by a trained psychometrician, using a standardized neuropsychological battery for cSLE [9]. Under consideration of age, race and gender, published norms were used to score the participants' performance on each of the formal neuropsychological tests, with results expressed as z-scores; these have a mean of 0 and standard deviation of 1 for a normative healthy population. Performance in each of the four cognitive domains assessed (working memory, psychomotor speed, attention, and visuoconstructional ability (VCA)) was quantified by averaging the z-scores of the standardized tests for each cognitive domain. Table 1 provides additional details about the standardized tests administered.

**Table 1:** Tests used to define neurocognitive dysfunction

Domain	Measure	Source	Description
**Working memory**	Digit span	Age-appropriate Wechsler intelligence scale [32,33]	Ability to repeat back in order, or in a re-sequenced order, increasingly difficult strings of numbers
	Letter-number sequencing	Age-appropriate Wechsler intelligence scale [32,33]	Ability to mentally re-sequence a series of letters and numbers before repeating them back
**Psychomotor speed**	Coding	Age-appropriate Wechsler intelligence scale [32,33]	Test-takers decode and transcribe a series of symbols as quickly as possible
	Symbol search	Age-appropriate Wechsler intelligence scale [32,33]	Score reflects speed and accuracy of test-takers' visual searches for matches in rows of symbols
**Attention**	Hit reaction time standard error	Conners' continuous performance test II [34]	On a 15-minute-long boring task, the variability in reaction time to specific letters flashing on screen
	Inhibition vs. color naming score	Delis-Kaplan executive functioning system [35]	Relative ability to focus on the color of the ink in which a conflicting color word is printed (for example, 'blue' written in red ink).
**Visuoconstructional abilities**	Block design	Wechsler abbreviated scales of intelligence [36]	Ability to efficiently reproduce colored line drawings using blocks with sides that have varying patterns
	Block counting	Kaufman assessment battery for children [37,38]	Ability to mentally represent the volume of a three-dimensional block construction printed in two-dimensional space

Participants with at least one domain z-score ≤ -2 or at least two domain z-scores ≤ -1 were categorized as members of the NCD-group. Otherwise, participants were considered members of the noNCD-group [10].

### Functional magnetic resonance imaging paradigms

During a single imaging session, each participant completed three fMRI paradigms probing attention, working memory, and VCA. Each of the fMRI paradigms used a block periodic design, with active task intervals interleaved with control task intervals. Visual stimuli were projected onto a screen 50 cm behind the subject's head and viewed by a mirror attached to the head coil. Presentation^® ^software (Neurobehavioral Systems, Inc.; Albany, CA, USA) running on a dedicated computer was used for stimulus display and response collection. Subjects responded using a handheld response box (Current Designs, Inc.; Philadelphia, PA, USA) that was connected by optical fiber to the computer. Each response was recorded and subsequently analyzed for correctness and response time.

#### Attention paradigm

Attention allows concentration on a specific target stimulus over a span of time, while avoiding distraction from extraneous stimuli. An identical pairs continuous performance task (CPT-IP), consisting of identifying the repetition of any item in a sequence, was utilized in this study to measure attention [11]. The active attentional task consisted of viewing a random single digit between 0 and 9 at the center of a screen every 0.75 seconds during a 30-second block. Participants were instructed to press a button whenever consecutive numbers were identical. During the control task intervals, also lasting 30 seconds, the number 1 was shown repeatedly with the same 0.75-second period. The participant was asked to press the response button five times at the beginning of this interval, with no further response required. The CPT-IP session was comprised of five interleaved blocks of each task type. The contrast between the attention and control tasks in this paradigm minimizes motor response from pressing buttons as well as visual stimulation associated with watching the numbers, allowing for the delineation of the attention needed to detect sequential pairs of numbers as they appear on the screen.

#### Working memory paradigm

Working memory allows for information to be maintained and available for use for a brief period of time. An N-back paradigm was used in this study to invoke working memory [11]. Participants performed a 2-back working memory task controlled for attention, visual stimulation, and motor response by a 0-back task. In both tasks, the integers from 1 to 4 were presented randomly, one at a time, on a screen with each number appearing consistently in a specific quadrant of a diamond shape. Patients responded by pressing buttons on a response box on which buttons had been arranged in a diamond pattern, corresponding spatially with the numbers appearing on the screen. During 30-second blocks, 17 numbers were presented at a steady rate. For the 2-back working memory task, subjects were instructed to press the button corresponding to the number that preceded the current number by two trials. The 0-back control task required the subject to merely press the button corresponding to the number currently showing on the screen. Since both tasks have the same sensorimotor and attentional elements, the contrast between them isolates the working memory component exclusive to the 2-back task.

#### Visuoconstruction paradigm

**Figure 1:**
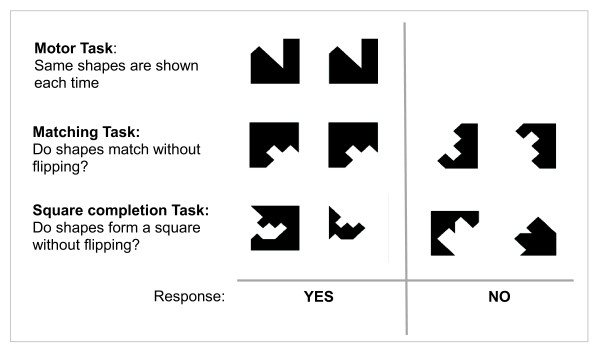
**The visuoconstruction paradigm consisted of three tasks for each of which the subject responded 'yes' or 'no' after each viewing of a pair of black shapes**. Example stimuli are shown for: the motor task (top row), for which the subject responded 'yes' to the same pair of matching shapes shown repeatedly; the matching task (middle row), for which 'yes' means the shapes match without flipping and 'no' means one is flipped, and the square completion task, for which subjects respond 'yes' for shapes that fit together to form a square without flipping and 'no' otherwise.

Visuoconstruction involves the ability to organize and manually manipulate spatial information to make a design. We adapted a previously developed fMRI paradigm to probe VCA in children [12,13]. This paradigm employed visual stimuli, each comprised of a pair of black geometric shapes on a white background, presented at regular time intervals. Stimuli were shown in blocks of 21 seconds duration, each introduced by 3 seconds of instructional text. Each block had the subject perform under one of three conditions: motor, matching, and square completion. Employing two buttons on the response box (corresponding to yes/no), the motor condition simply required pressing the yes-button after each presentation of the same pair of identical shapes (Figure 1). During the matching condition, the subject responded 'yes' if the shapes were identical, or 'no' if one of the shapes was flipped relative to the other (Figure 1). For the square completion condition participants were asked to judge if the two shapes could be fitted together to form a complete square, again without flipping either shape, although shapes might have to be rotated. One run of the VCA paradigm consisted of eighteen blocks; six motor, six matching, and six square completion conditions, with the block order pseudo-randomized and balanced. The motor condition provides control for motor, attention, and primary visual elements of the matching and square completion conditions.

While the contrasts of matching vs. motor conditions and square completion vs. motor conditions both delineate brain activation involving visuoconstruction, the contrast between the square completion vs. matching conditions further controls for purely perceptual function to focus on constructional ability [12].

### Magnetic resonance imaging

Imaging was performed at two separate sites with matching protocols using a Philips Achieva 3 Tesla (3T) and a Siemens Trio 3T scanner, respectively. The same T2*-weighted gradient-echo echo-planar imaging sequence was used for all fMRI paradigms, with the following parameters: repetition time (TR) 3,000 ms (milliseconds), echo time (TE) 30 ms, field of view (FOV) 256 × 256 mm, matrix 64 × 64 pixels, 44 axial slices, slice thickness 3 mm. In addition, a high-resolution, T1-weighted, inversion-prepared three-dimensional magnetization-prepared rapid gradient echo (MPRAGE) whole brain scan was acquired for each study participant. Parameters for this scan were as follows at site 1: TR 6.8 ms, TE 2.9 ms, inversion recovery time 904 ms, FOV 176 × 256 × 256 mm, matrix 176 × 256 × 256 pixels (total time 6 minutes, 42 seconds). At site 2, the parameters were identical except for a TE of 3.1 ms and an inversion recovery time of 900 ms to account for differences in scanners. These scans served as the anatomic reference for co-registration and overlay of functional data. During each session, the MPRAGE volume was acquired first, followed by the functional imaging. A series of 148 images (total time 7 minutes, 24 seconds) was acquired for the VCA paradigm, while the CPT-IP and N-back tasks required 114 images each (total time 5 minutes, 42 seconds). Data from the initial four time points were discarded from the imaging series of each paradigm to allow for attainment of T1 relaxation equilibrium.

In order to compare signal characteristics between sites and to monitor stability, a phantom was scanned each day a subject was imaged, as recommended for multisite studies by the Function Biomedical Informatics Research Network (FBIRN) [14]. Matching phantoms, obtained from an FBIRN source, were used at each site, comprised of a 17.5 cm-diameter spherical plastic shell filled with a solution of nickel chloride and sodium chloride in agar. The solution is proportioned to load the MR coil like a human head and to have relaxation characteristics similar to human gray matter at 3 Tesla. Phantom series included 200 images using parameters identical to those employed for fMRI sessions. In total, fifteen phantom series were completed on the Philips scanner and five were completed on the Siemens scanner.

#### Image processing

Processing of three-dimensional anatomic and fMRI data was done using Statistical Parametric Mapping (SPM) software [15] in the Matlab computing environment (The Mathworks, Inc., Natick, MA, USA). Prior to statistical analysis, several preprocessing steps were completed: 1) rigid-body realignment of each image to the first image of each session, using three translational and three rotational adjustments; 2) co-registration of the session mean functional image to the corresponding anatomical image; 3) normalization to Montreal Neurological Institute (MNI) template space, and 4) smoothing with an 8-mm full width at half maximum (FWHM) Gaussian kernel. Transformation parameters for normalization resulted from anatomical segmentation in SPM8 based on gray matter, white matter, and cerebral spinal fluid templates. Application of the same transformation to the corresponding preprocessed functional images permitted overlay of statistical parametric maps onto the anatomic reference and allowed the voxel-by-voxel combination of data from multiple subjects into a group activation map for each fMRI task.

Phantom data were analyzed via dedicated software obtained from FBIRN [16]. Site comparison for this study focused on calculations of signal-to-noise ratio (SNR) and signal-to-fluctuation noise ratio (SFNR).

### Statistical analysis

Continuous measures of sociodemographic and cSLE relevant information as well as numbers of correct responses during each of the fMRI tasks were summarized by means and SDs and compared using 2 sample *t*-tests between groups. For categorical variables, frequencies (in %) of correct responses per fMRI task were compared using Fisher's exact test.

The functional imaging data were analyzed using both voxel-wise- and region of interest (ROI)-based approaches. Voxel-based analyses are able to examine activations across the entire brain, but are limited by the need for more stringent correction for multiple comparisons, and thus, are less sensitive. ROI-based analyses examine neuronal activation only in pre-specified regions of the brain linked to a given fMRI task. ROI-based analyses sacrifice examining the entire brain for enhanced sensitivity to capture group differences at the selected brain areas using aggregate measures.

#### Voxel-wise analysis

Voxel-wise brain activation was compared between the NCD and noNCD-groups using the following two-level analytic approach. At the first level, brain activation was estimated for each fMRI task at each voxel under the general linear model framework. The design matrix included the block periodic time series for each condition of the task. The time series was adjusted for the known delay of BOLD responses using the canonical hemodynamic response function in SPM8. Motion parameters were included as nuisance covariates in the design. Contrasts of interest between task conditions were assessed as differences in corresponding estimated model parameters. In the second level, a random- (or mixed-) effect model was used to compare means between groups at each voxel, after accounting for within-person (or between-voxel) relationships using a random effect. Phantom data suggested differences between scanners used in this study. While SFNR was markedly similar between scanners (Philips = 271 ± 5 vs. Siemens = 263 ± 17, *P *= 0.37), SNR differed significantly (Philips = 314 ± 31 vs. Siemens = 272 ± 33, *P *= 0.05). In light of this, the analysis was adjusted for a site effect by adding a site covariate to the model. Differences in mean activation between groups were assessed for each task contrast separately using the two-sample *t*-test. The resulting T-score maps were thresholded at a nominal voxel *P*-value of 0.005, uncorrected for multiple comparisons, with resulting clusters of voxels assessed in SPM8 for significance at a corrected *P*-value < 0.05.

#### Region of interest analysis

All ROIs were chosen prior to analysis based on previously published reports of activation of fMRI tasks for working memory [17], attention [18], and VCA [19] similar to those employed in this study. In addition, a collection of regions that commonly deactivate during task performance, known as the default mode network, were identified using previous reports [20]. ROIs were extracted from the automated anatomical labeling (AAL) atlas stored in the Wake Forest University Pick-Atlas toolbox in SPM8. A list of the ROIs considered in this analysis for each task is provided in Table 2.

**Table 2:** Regions of interest considered per functional magnetic resonance imaging task

Domain: Task	Regions of interest
**Working memory:** **N-back**	Frontal, middle + inferior
	Anterior cingulate
	Precuneus
	Parietal, inferior
	Anterior default mode (medial prefrontal cortex) ^1^
	Precuneus + posterior cingulate^1^
	Angular gyrus^1^
	Hippocampus + parahippocampus^1^
	Temporal, superior^1^
**Attention:** **CTP-IP**	Frontal, inferior
	Frontal, middle
	Insula + temporal, superior
	SMA + cingulate, middle
	Parietal, inferior + supramarginal gyrus
	Fusiform gyrus + occipital, inferior
	Frontal mid, inferior + precentral gyrus
	Default, anterior (medial prefrontal cortex) ^1^
	Precuneus + posterior cingulate^1^
	Angular gyrus^1^
	Hippocampus + parahippocampus^1^
**Visuo-construction:** **VCA**	Frontal, inferior
	Frontal, middle
	Parietal, Inferior + supramarginal gyrus
	Fusiform gyrus + occipital, inferior
	Precuneus, bilateral
	Parietal, superior
	Frontal, superior
	Frontal, superior medial bilateral
	Default, anterior (medial prefrontal cortex)^1^
	Precuneus + posterior cingulate^1^
	Angular gyrus^1^
	Hippocampus + parahippocampus^1^

All regions chosen prior to analyses and derived from previously published reports (references [15-18]). ^1^Regions hypothesized to deactivate under the task; otherwise, the region is hypothesized to activate. Unless otherwise indicated, right and left hemisphere portions of each region are considered separately in analyses. CPT-IP, continuous performance task-identical pairs; VCA: visuoconstructive ability; SMA, supplementary motor area.

For each ROI, its activation (or deactivation) level was measured by aggregating T-scores [21,22] from activated (or deactivated) voxels that fit the following two criteria: (1) those T-scores were all above (or below) a threshold of *t *≥ 1.64, corresponding to a one-sided significance level of 0.05, and (2) the voxels had to be part of a cluster of at least 10 activated (or deactivated) adjacent voxels. This threshold and cluster size within a given ROI was chosen to help reduce noise, hence avoid the detection of spurious activations in isolated voxels. Use of aggregate T-scores provides a measure that reflects a combination of activation and variance.

For each task-specific ROI, the association between brain activation and NCD status was determined using an analysis of variance (ANOVA) model, after controlling for imaging site. Post hoc means of activation were compared between the NCD-group and the no-NCD-group under the ANOVA model framework.

The relationship of each domain z-score from formal neuropsychological testing and the ROI activation level was determined by calculation of a partial correlation coefficient, after controlling for imaging site. All analyses were repeated after adding more controlling covariates, such as family income levels and the current dose of systemic steroids to the models. Because the results of models with these additional covariates were found no different from those adjusting only for imaging site, they are not reported. All ROI analyses were performed using SAS 9.3 software (SAS, Cary, NC, USA). *P*-values < 0.05 were considered statistically significant.

## Results

### Patients

Among the twenty-two study participants, fourteen had normal cognition (noNCD-group) and eight were found to have NCD (NCD-group) based on their performance during formal neuropsychological testing. One participant with NCD failed to complete the fMRI session and was excluded, leaving a total of twenty-one participants, including seven with NCD, with complete data. Key demographic and cSLE-related data from the study population are summarized in Table 3. As expected, children with NCD has significantly lower intelligence quotient (IQ) scores and were exposed to higher daily doses of corticosteroids compared to children with normal cognition. There was a trend towards a higher socioeconomic level as measured by the highest maternal educational level in families of children with normal cognition. At the time of the study the NCD-group was treated with significantly higher doses of prednisone than in the noNCD-group, and the annual income of the NCD-group was significantly lower. Formal neuropsychiatric testing revealed that the domain z-scores for psychomotor speed, working memory, and VCA were significantly lower in the NCD-group than the noNCD-group. Conversely, groups only showed small and statistically insignificant differences in the domain z-scores for attention during formal neuropsychological testing.

**Table 3:** Demographics of study participants

		cSLE without NCD (*n *= 14)	cSLE with NCD (*n *= 7)	*P-*value^1^
**Age, years**		14.7 ± 2.1	15.1 ± 1.9	NS
**Female**	Number, %	11 (78.6%)	6 (85.7%)	NS
**Race/ethnicity**	White/black/hispanic/other	6/4/2/2	1/6/0/0	NS
**Highest maternal educational level**	Postgraduate degree/Bachelor's degree/partial college or associate degree/High School degree/unknown	2/4/4/4/0	0/0/3/3/1	NS
**WASI full scale IQ score**		104.4 ± 10.5	89.0 ± 7.4	0.005
**Annual family income (in "tabcaption",000)**		84.4 ± 48.9	34.4 ± 17.3	0.003
**Disease duration, years**		2.5 ± 2.2	1.6 ± 1.6	NS
**Medications**	Prednisone (mg/kg/day)	11.7 ± 7.4	16.9 ± 11.8	NS
	Treatment with immunosuppressant^2^	5 (35.7%)	5 (71.4%)	NS
**Disease activity and damage**	SLEDAI score	4.1 ± 3.0	7.1 ± 6.2	NS
	SDI score	0.4 ± 0.8	0.7 ± 1.1	NS
**Imaging sites**	Site 1/site 2	10/4	6/1	NS
**Neurocognitive dysfunction**	Average z-scores			
	Working memory	-0.15 ± 0.52	-1.03 ± 0.59	0.0038
	Psychomotor speed	0.23 ± 0.69	-1.22 ± 0.38	0.0001
	Attention	0.13 ± 0.57	-0.39 ± 1.08	NS
	Visuoconstructional ability	0.30 ± 0.46	-1.22 ± 0.91	< 0.0001

Values are means and SD unless indicated otherwise. ^1^*P*-values are based on *t*-tests or Fisher's exact test, when appropriate. ^2^Number of patients (without/with) neurocognitive dysfunction (NCD) treated with: mycophenolate mofetil (4/2); azathioprine (2/1); methotrexate (1/0); mycophenolate mofetil plus cyclophosphamide (0/1); cyclophosphamide (0/1). cSLE, childhood-onset systemic lupus erythematosus; WASI, Wechsler abbreviated scales of intelligence; NS, not significant; SLEDAI, Systemic Lupus Erythematosus Disease Activity Index (2-k version; range 0 to 105; 0 = inactive disease); SDI: Systemic Lupus International Collaborating Clinics/American College of Rheumatology Damage Index (range 0 to 46; 0 = no damage).

### Performance on fMRI tasks in the scanner

The correct response rates and response times required for correct responses during the CPT-IP attention, 2-back working memory, and VCA fMRI tasks were compared between groups. The mean response times for all three fMRI tasks were not significantly different between groups. Conversely, compared to the noNCD-group, the NCD-group had a significantly lower correct response rate for the 2-back working memory fMRI task (25% ± 15% for the NCD-group vs. 64% ± 22% for the noNCD-group; *P *< 0.0003) and the square completion condition that is part of the VCA fMRI task (47% ± 12% for the NCD-group vs. 63% ± 19% for the noNCD-group; *P *< 0.03). The correct response rate for the CPT-IP attention fMRI task, however, did not differ significantly between groups (71% ± 29% for the NCD-group vs. 85% ± 21% for the noNCD-group; *P *< 0.29). Control tasks for both N-back and VCA showed improved performance with insignificant group differences: 0-back (82% ± 21% for the NCD-group vs. 90% ± 21% for the noNCD-group; *P *< 0.42), VCA matching condition (63% ± 18% for the NCD-group vs. 74% ± 23% for the noNCD-group; *P *< 0.22).

### Neuronal network activation

#### Voxel-wise analysis

**Figure 2:**
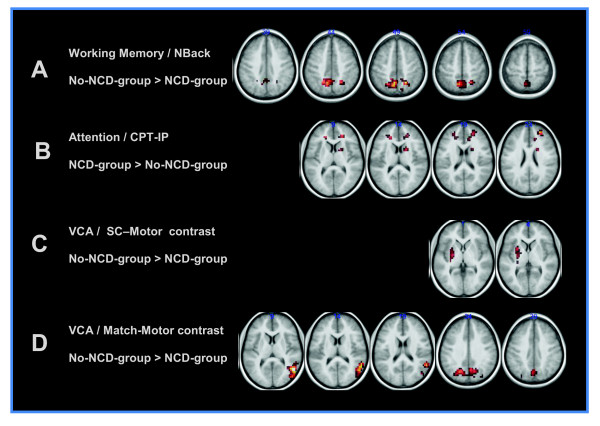
**Voxel-wise differences in functional neural network activations during functional magnetic resonance imaging**. Differences in neuronal network activation between participants with neurocognitive dysfunction (NCD) (NCD-group; *n *= 7) and participants without NCD (noNCD-group; *n *= 14) are depicted. The clusters shown reflect differences between groups of participants at an uncorrected *P*-value < 0.005 and cluster size threshold of 40 voxels. Neurological convention of image orientation is used. (**A**) Clusters in which the noNCD-group activated more strongly than the NCD-group for the N-Back working memory paradigm. (**B**) Continuous performance task-identical pairs (CPT-IP) (attention) displays clusters with the NCD-group activating more strongly than the no-NCD-group. Visuoconstructional ability (VCA) task differential activations with the noNCD-group showing more activation than the NCD-group for (**C**) the square completion (SC) vs. motor and (**D**) the matching vs. motor contrasts.

T-maps of differences in activation between the NCD-group and the noNCD-group for the three paradigms are displayed in Figure 2 at nominal thresholds of *P *< 0.005, uncorrected for multiple comparisons, and a voxel cluster size > 40. The working-memory task produced differences in a cluster of voxels located in the precuneus, extending into the inferior parietal regions, for which the noNCD-group had significantly more brain activation than the NCD-group (*P *< 0.05 for the entire cluster, after correction for multiple comparisons between groups). The VCA paradigm also produced stronger activation in the noNCD-group compared to the NCD-group, with significant clusters (corrected cluster *P*-value < 0.05) in the precuneus and right occipital regions for the matching vs. motor contrast of the VCA paradigm. There were also trends in differential brain activation in the basal ganglia between groups for the square completion vs. motor contrast. The square completion vs. matching contrast failed to show activation at the uncorrected *P *< 0.005 threshold for individual voxels. While the NCD-group exhibited lower brain activation during the working memory and VCA fMRI tasks than the noNCD-group in relevant brain regions, there was a trend toward greater brain activity in the frontal lobe regions in the NCD-group than in the noNCD-group during the CPT-IP attention task.

#### ROI-based analysis

In line with the voxel-wise analysis, ROI-based analysis suggested significant differences between groups in neuronal network activation in select ROIs that are considered relevant for the N-back, VCA and CPT-IP fMRI tasks (Table 4). The noNCD-group consistently activated several ROIs relevant for the N-back and VCA fMRI tasks more strongly than the NCD-group. Conversely, during the CPT-IP attention fMRI task, brain activation in the left insular/superior temporal region was more pronounced in the NCD-group than the noNCD-group. There were no significant differences between groups in any of the ROIs expected to deactivate during task execution of the N-back working memory, CTP-IP attention and VCA fMRI tasks. Details of group activation comparisons for all ROIs are available as supplementary material (Additional file 1).

**Table 4:** Regions of interest with significant differences in activation between cSLE patients with versus without neurocognitive dysfunction

	Anatomical region of interest	no-NCD group	NCD group	p-value
**Working memory/N-back**	Precuneus	1.05 ± 0.17	0.13 ± 0.25	0.004
	Parietal inf L	2.32 ± 0.32	1.10 ± 0.47	0.030
	Parietal inf R	3.22 ± 0.41	1.77 ± 0.59	0.041
**Attention/CPT-IP**	Insula + temporal sup L	0.45 ± 0.19	1.06 ± 0.27	0.050
**VCA/match-motor contrast**	Frontal inf L	0.71 ± 0.12	0.25 ± 0.17	0.032
	Frontal mid L	0.72 ± 0.12	0.26 ± 0.18	0.029
	Frontal sup L	0.50 ± 0.10	0.15 ± 0.14	0.037
	Parietal inf +supramarginal L	1.19 ± 0.16	0.66 ± 0.23	0.050
	Parietal sup L	1.62 ± 0.23	0.81 ± 0.34	0.045
	Fusiform+occipital inf L	1.58 ± 0.27	0.59 ± 0.40	0.038
	Fusiform+occipital inf R	1.49 ± 0.26	0.52 ± 0.39	0.036
**VCA/square-motor contrast**	Frontal sup R	0.54 ± 0.09	0.21 ± 0.13	0.033
	Precuneus bilateral	0.85 ± 0.14	0.34 ± 0.20	0.036
	Fusiform+occipital inf L	1.95 ± 0.24	1.06 ± 0.35	0.033
	Fusiform+occipital inf R	1.54 ± 0.22	0.77 ± 0.33	0.048

Region of interest (ROI) activation is defined as the (sum of T-scores within the ROI among voxels with T-scores > 1.66 that are part of clusters of at least 10 voxels)/total voxels in the ROI. cSLE, childhood-onset systemic lupus erythematosus; NCD, neurocognitive dysfunction; CPT-IP: continuous performance task- identical pairs; VCA, visuoconstructive ability; L, left; R, right; inf, inferior; mid, middle.

We then assessed the relationship between participant performance during formal neuropsychological testing and neuronal network activation patterns. Table 5 presents statistically significant partial correlation coefficients (after adjusting for imaging site) between brain activation and neuropsychological domain scores (attention, working memory, VCA, and processing speed). It is worth noting that although each standardized test primarily challenged a particular cognitive domain, successful performance also relied on an array of other cognitive skills. Therefore, a complex pattern of relationships was observed between brain activation during a given fMRI task and the cognitive performance measured on neuropsychological tests. As might be expected, brain activation of the ROIs stimulated by the attention (CPT-IP) and VCA fMRI tasks were correlated with the attention and VCA domain z-scores. Unexpectedly, working memory performance during neuropsychological testing was not associated with activation in any of the ROIs for the N-back working memory fMRI task. Instead, it was found to be negatively correlated with CPT-IP activation in several ROIs.

**Table 5:** Summary of association between region of interest activity and cognitive domain z-scores under formal neuropsychological testing

fMRI paradigm	Region of interest	Attention domain	VCA domain	Working memory domain
**Working memory: N-back**	Frontal mid inf R	0.51		
	Precuneus	0.47	0.54	
	Parietal inf R		0.45	

**Attention: CTP-IP**	Frontal mid L			-0.45
	Insula + temporal sup L			-0.46
	SMA + cingulate mid bilateral			-0.47
	Fusiform +occipital inf L	0.52		

**VCA: match vs. motor contrast**	Frontal sup L		0.45	
	Parietal sup L		0.48	
	Fusiform + occipital inf R		0.45	
	Angular R			0.51

**VCA: SC vs. motor contrast**	Frontal mid L		0.45	
	Frontal sup L		0.64	
	Frontal sup R	0.49	0.65	
	Precuneus bilateral	0.54	0.62	
	Parietal sup L	0.50	0.56	
	Parietal sup R	0.48		
	Fusiform + occipital inf L		0.52	
	Fusiform + occipital inf R		0.48	

Values are partial correlation coefficients (*r*-values). Only *r*- values > 0.45 are shown, corresponding to *P *< 0.05. fMRI, functional magnetic resonance imaging; CPT-IP: continuous performance task-identical pairs; VCA, visuoconstructive ability; SC, square completion; L, left; R, right; inf, inferior; sup, superior; mid, middle; SMA, supplementary motor area.

## Discussion

We found differential neuronal activation in several brain regions during fMRI tasks exercising VCA, working memory, and attention in children with cSLE who have NCD, compared to those with normal cognition based on formal neuropsychological testing. Furthermore, we newly report details about the relationship between the level of cognitive performance and task-driven regional brain activation in cSLE.

Previous investigations have reported various cognitive deficits in both adult SLE and cSLE, most commonly including impairment of attention, working memory, and VCA [23-25]. Earlier work applying fMRI in adults and children explored changes in neuronal networks associated with SLE in comparison to healthy controls [11,26-30]. These studies suggest that individuals with SLE activate brain regions associated with specific cognitive tasks more strongly than their healthy counterparts. The recruitment of the additional brain regions was hypothesized to help maintain normal levels of performance by patients with cSLE or adults with SLE during a given fMRI task.

The results of our study support the use of a similar compensatory mechanism for NCD for the attention task CPT-IP. We demonstrated greater activation in an insular/superior temporal ROI in the presence of clinically overt NCD compared to no NCD while CPT-IP performance remained unaffected. Notably, a negative relationship between CPT-IP brain activation in several ROI and patients' working memory abilities, as measured by formal neuropsychological testing, was observed. A possible explanation for this finding is that CPT-IP is, in effect, a 1-back working memory task. It represents a simpler version of the N-back (2-back) task. Lack of negative association of CPT-IP activation with attention domain scores suggests that attention deficits may not be the principal drivers of compensatory activation for this task. The current study in young patients with cSLE directly supports the notion that there is a relationship between the presence of NCD and alterations in brain activation.

Mackay *et al*. reported that longer SLE disease durations are associated with diminished neuronal activation during an fMRI task of working memory among adults with SLE [28]. The patients included in that study [28] with longer disease durations also had more disease damage and poorer working memory, making it difficult to dissect causal relationships. Conversely, in our study the NCD and noNCD groups had similar disease duration, suggesting that the duration of SLE may not be as important for brain activation differences in SLE as previously thought. Alternatively, the differences in findings between our study and Mackay's might be due to differences in how developing brains of children with cSLE and mature brains of adults with SLE respond to the underlying inflammatory processes.

Nonetheless, the observations by Mackay *et al*. suggest that compensatory augmentation of task activation can break down with sufficient levels of disease damage. It is plausible that the threshold for breakdown also depends on task difficulty. Unlike the CPT-IP task, we observed diminished activation in select brain regions pertaining to the N-back and VCA tasks in cSLE patients with NCD compared to those with no NCD. Thus, some neuronal activity changes we observed in children with cSLE-related NCD parallel those in adults with SLE of extended duration. Intriguingly, this decrease of activation associated with NCD occurred in the precuneus and inferior parietal areas, which are regions where cSLE patients have previously showed more brain activation than healthy controls under a working memory fMRI task [11].

In aggregate, our results are consistent with a model in which intact cognitive performance can be maintained in children and adults with SLE via compensatory increased neuronal activation. However, under sufficient disease burden or cognitive challenge, that compensatory pattern breaks down, resulting in diminished activation and clinically apparent cognitive dysfunction.

As a group, the children with NCD in this study had a significantly lower correct response rate than the noNCD-group during fMRI tasks probing working memory and VCA, while performance on the CPT-IP attention fMRI task showed no significant group differences. Correspondingly, while activation decreased for working memory and VCA in those with NCD, the attention task (CPT-IP) elicited stronger activations for the NCD-group on both voxel-based and ROI-based analyses. It is possible that the CPT-IP task did not challenge the study participants as much as the other fMRI tasks, allowing the NCD-group to maintain performance by means of the compensatory strategy of greater activation described above. Alternatively, the lack of CPT-IP performance differences between the NCD-group and the noNCD-group may be due to relatively well-matched attention domain z-scores in formal neuropsychological testing, or to a more robust mechanism for maintaining attentional ability compared to working memory function or VCA.

For all three fMRI tasks, there is a common thread of positive correlation between activation and attention domain formal neuropsychological testing z-scores. Thus attention deficits in the NCD-group may play a role in diminishing performance during fMRI tasks that probe working memory and VCA, and the corresponding decrease in brain activation in patients with NCD.

This study offers a framework for viewing NPSLE as a burden on brain function that elicits compensatory mechanisms, relying on neuronal plasticity to maintain cognitive performance. Plasticity has limits which, when exceeded result in clinical manifestations of cognitive deficit. In the context of brain plasticity, the course of NPSLE may have a more profound impact on children with cSLE, given their dramatic, ongoing, brain development. While children possess a greater capacity for brain plasticity in response to an assault such as NPSLE [31], there remains the potential for the disease not only to disrupt existing neuronal networks, as demonstrated in this study, but also to alter the development of emerging brain networks. Our cross-sectional study does not allow us to delineate whether brain development is impaired by cSLE, or whether ongoing brain maturation serves as a means to help compensate for earlier brain insults by active NPSLE. Such a critical unknown will require longitudinal studies, which will also help determine whether the observed alterations in brain activation are permanent or reversible with treatment.

A distinct strength of our study is that its participants were well-phenotyped with respect to their clinical cSLE status, and their cognitive abilities were accurately assessed using the cSLE Cognitive Battery of Standardized Tests [9]. However, this study also suffered from some limitations. While twenty-one subjects were imaged for this study, only seven tested as having NCD by our criteria. This low number limited the statistical power for finding differences attributable to the development of NCD. Nonetheless, significant differences between groups of children with different levels of cognitive ability were detected, and one might speculate that at least some of the trends in associations would have reached statistical significance had the sample size been larger. Performance on both 2-back and VCA square completion fMRI tasks was particularly poor for the NCD group raising concern about motivation. This is dispelled to some degree by good performance by the NCD group on the CPT-IP task and the control tasks for N-back and VCA. Recruitment for this study included children in the age range of 9 to 18 years, a period of ongoing brain development. Including this span of development may have introduced age-related variability in the fMRI findings. Note, however, that the NCD and noNCD groups are well matched in age, reducing the impact of developmental stage on group difference assessments. The subject groups were not matched, however, in socioeconomic status (SES) and IQ, with the NCD group lower in both measures. The influence of these differences on our results cannot be ruled out.

## Conclusions

In summary, we found differences in brain activation patterns that are related to distinct cognitive deficits in cSLE. Building on the results of our previous study [6], we postulate that cSLE leads to changes in brain function, which are initially compensated by increased activation in certain brain areas; once compensatory mechanisms fail, clinically overt NCD occurs. Further research is required to delineate brain activation correlates associated with the resolution of clinically overt NCD and those with persistent cognitive deficits in SLE.

## Abbreviations

AAL: automated anatomical labeling; ANOVA: analysis of variance; BOLD: blood oxygenation level-dependent; CPT-IP: continuous performance task-identical pairs; cSLE: childhood-onset SLE; FBIRN: Function Biomedical Informatics Research Network; fMRI: functional magnetic resonance imaging; FOV: field of view; FWHM: full width at half maximum; IQ: intelligence quotient; MNI: Montreal Neurological Institute; MPRAGE: magnetization-prepared rapid gradient echo; NCD: neurocognitive dysfunction; NPSLE: neuropsychiatric systemic lupus erythematosus; ROI: region of interest; SES: socioeconomic status; SFNR: signal-to-fluctuation noise ratio; SLE: systemic lupus erythematosus; SNR: signal-to-noise ratio; SPM: Statistical Parametric Mapping;TE: echo time; TR: repetition time; VCA: visuoconstructional ability.

## Competing interests

The authors declare that they have no competing interests.

## Authors' contributions

HB, MD, and DG participated in study design. MD, DG, TP, and AS-G took part in imaging data acquisition. MK-G, FZ, DB, HB, and AS-G carried out neuropsychological data acquisition. MD and JY performed data analysis. MD, DG, JY, and HB participated in interpretation of results. MD, HB, and JY prepared the manuscript. All authors participated in review and approval of the final manuscript.

## Supplementary Material

Additional file 1 **Summary of brain activity vs. NCD status for all regions of interest examined**. A table listing mean region of interest (ROI) activation, defined as (sum of T-scores within the ROI among voxels with T-scores > 1.66 that are part of clusters of at least 10 voxels)/total voxels in the ROI, for systemic lupus erythematosus (SLE) patients with and without neurocognitive dysfunction (NCD). Click here for file
