# Supplementary material for: Functional neuronal network activity differs with cognitive dysfunction in childhood-onset systemic lupus erythematosus
Source: Arthritis Res Ther. 2013 Mar 7;15(2):R40. doi: 10.1186/ar4197 (PMC3672728; doi:10.1186/ar4197)
Supplement: Additional file 1 — Summary of brain activity vs. NCD status for all regions of interest examined. A table listing mean region of interest (ROI) activation, defined as (sum of T-scores within the ROI among voxels with T-scores > 1.66 that are part of clusters of at least 10 voxels)/total voxels in the ROI, for systemic lupus erythematosus (SLE) patients with and without neurocognitive dysfunction (NCD). [file ar4197-S1.DOCX]

| **Table -Summary of brain activity vs. NCD status for all Regions of Interest examined**** | | | | | |
| --- | --- | --- | --- | --- | --- |
| **Test** | | **ROI** | **Mean ± SE** | | **p-value** |
|  |  |  | **SLE**  **without NCD** | **SLE with**  **NCD** |  |
| **Working Memory:**  **N-Back** | 1.Frontal, Mid+Inf L | | 1.92 ± 0.28 | 1.17 ± 0.41 | 0.120 |
|  | 2.Frontal, Mid+Inf R | | 2.28 ± 0.33 | 1.28 ± 0.47 | 0.074 |
|  | 3.ACC | | 0.27 ± 0.12 | 0.45 ± 0.18 | 0.369 |
|  | **4.Precuneus** | | **1.05 ± 0.17** | **0.13 ± 0.25** | **0.004** |
|  | **5.Parietal Inf L** | | **2.32 ± 0.32** | **1.10 ± 0.47** | **0.030** |
|  | **6.Parietal Inf R** | | **3.22 ± 0.41** | **1.77 ± 0.59** | **0.041** |
|  | *7.Anterior Default mode (mPFC)* | | -1.21 ± 0.23 | -0.71 ± 0.34 | 0.201 |
|  | *8.Precuneus +PCC* | | -1.01 ± 0.20 | -1.09 ± 0.29 | 0.810 |
|  | *9.Angular L* | | -0.68 ± 0.22 | -0.88 ± 0.32 | 0.566 |
|  | *10.Angular R* | | -0.26 ± 0.07 | -0.20 ± 0.11 | 0.580 |
|  | *11.Hippocampus/parahippocampus L* | | -1.24 ± 0.20 | -1.00 ± 0.29 | 0.467 |
|  | *12.Hippocampus/parahippocampus R* | | -1.11 ± 0.19 | -0.87 ± 0.28 | 0.450 |
|  | *13.Temporal Sup L* | | -1.46 ± 0.32 | -1.58 ± 0.46 | 0.813 |
|  | *14.Temporal Sup R* | | -1.25 ± 0.29 | -1.43 ± 0.42 | 0.705 |
| **Attention:**  **CTP-IP** | 1.Frontal Inf L | | 0.94 ± 0.22 | 1.34 ± 0.32 | 0.279 |
|  | 2.Frontal Inf R | | 1.21 ± 0.35 | 1.84 ± 0.52 | 0.282 |
|  | 3.Frontal Mid L | | 0.93 ± 0.22 | 1.35 ± 0.32 | 0.248 |
|  | 4.Frontal Mid R | | 1.12 ± 0.36 | 1.72 ± 0.52 | 0.314 |
|  | ***5.Insula + Temporal Sup L*** | | ***0.45 ± 0.19*** | ***1.06 ± 0.27*** | ***0.050*** |
|  | 6.Insula + Temporal Sup R | | 0.49 ± 0.20 | 0.98 ± 0.28 | 0.140 |
|  | 7.SMA+Cingulate Mid bilateral | | 0.59 ± 0.22 | 1.21 ± 0.31 | 0.091 |
|  | 8.Parietal Inf + Supramarginal L | | 1.05 ± 0.25 | 1.35 ± 0.36 | 0.464 |
|  | 9.Parietal Inf + Supramarginal R | | 0.97 ± 0.25 | 1.35 ± 0.37 | 0.366 |
|  | 10.Fusiform+Occipital Inf L | | 1.82 ± 0.24 | 1.38 ± 0.34 | 0.272 |
|  | 11.Fusiform+Occipital Inf R | | 1.45 ± 0.26 | 1.29 ± 0.38 | 0.710 |
|  | 12.Frontal Mid Inf+Precentral L | | 1.02 ± 0.24 | 1.44 ± 0.34 | 0.281 |
|  | 13.Frontal Mid Inf+Precentral R | | 0.92 ± 0.29 | 1.50 ± 0.43 | 0.235 |
|  | *14.Default Ant (mPFC)* | | -0.60 ± 0.19 | -0.48 ± 0.28 | 0.714 |
|  | *15.Precuneus+PCC* | | -0.74 ± 0.25 | -0.75 ± 0.36 | 0.989 |
|  | *16.Angular L* | | -0.47 ± 0.20 | -0.49 ± 0.29 | 0.937 |
|  | *17.Angular R* | | -0.29 ± 0.14 | -0.50 ± 0.21 | 0.372 |
|  | *18.Hippocampus/parahippocampus L* | | -0.35 ± 0.13 | -0.45 ± 0.18 | 0.626 |
|  | *19.Hippocampus/parahippocampus R* | | -0.31 ± 0.11 | -0.33 ± 0.17 | 0.897 |
| **VCA:**  **Match/Motor contrast** | **1.Frontal Inf L** | | **0.71 ± 0.12** | **0.25 ± 0.17** | **0.032** |
|  | 2.Frontal Inf R | | 0.94 ± 0.17 | 0.42 ± 0.24 | 0.073 |
|  | **3.Frontal Mid L** | | **0.72 ± 0.12** | **0.26 ± 0.18** | **0.029** |
|  | 4.Frontal Mid R | | 0.93 ± 0.20 | 0.28 ± 0.29 | 0.063 |
|  | ***5.Parietal Inf +Supramarginal L*** | | ***1.19 ± 0.16*** | ***0.66 ± 0.23*** | ***0.050*** |
|  | 6.Parietal Inf+Supramarginal R | | 0.97 ± 0.16 | 0.57 ± 0.23 | 0.137 |
|  | **7.Fusiform+Occipital Inf L** | | **1.58 ± 0.27** | **0.59 ± 0.40** | **0.038** |
|  | **8.Fusiform+Occipital Inf R** | | **1.49 ± 0.26** | **0.52 ± 0.39** | **0.036** |
|  | *9.Default Ant (mPFC) bilateral* | | -0.25 ± 0.11 | -0.47 ± 0.16 | 0.252 |
|  | *10.Precuneus+PCC bilateral* | | -0.33 ± 0.11 | -0.45 ± 0.15 | 0.478 |
|  | *11.Angular L* | | -0.37 ± 0.22 | -0.65 ± 0.32 | 0.436 |
|  | *12.Angular R* | | -0.35 ± 0.16 | -0.64 ± 0.23 | 0.263 |
|  | *13.Hippocampus/parahippocampus L* | | -0.16 ± 0.07 | 0.00 ± 0.10 | 0.158 |
|  | *14.Hippocampus/parahippocampus R* | | -0.11 ± 0.05 | -0.14 ± 0.07 | 0.756 |
|  | 15.Precuneus bilateral | | 0.62 ± 0.16 | 0.14 ± 0.23 | 0.082 |
|  | **16.Parietal Sup L** | | **1.62 ± 0.23** | **0.81 ± 0.34** | **0.045** |
|  | 17.Parietal Sup R | | 1.43 ± 0.27 | 1.07 ± 0.39 | 0.416 |
|  | **18.Frontal Sup L** | | **0.50 ± 0.10** | **0.15 ± 0.14** | **0.037** |
|  | 19.Frontal Sup R | | 0.54 ± 0.14 | 0.16 ± 0.21 | 0.118 |
|  | 20.Frontal Sup Medial bilateral | | 0.43 ± 0.12 | 0.19 ± 0.18 | 0.254 |
| **VCA:**  **Square completion/ Match Contrast** | 1.Frontal Inf L | | 0.13 ± 0.08 | 0.22 ± 0.11 | 0.532 |
|  | 2.Frontal Inf R | | 0.16 ± 0.08 | 0.24 ± 0.12 | 0.564 |
|  | 3.Frontal Mid L | | 0.12 ± 0.11 | 0.27 ± 0.15 | 0.394 |
|  | 4.Frontal Mid R | | 0.15 ± 0.10 | 0.23 ± 0.14 | 0.654 |
|  | 5.Parietal Inf +Supramarginal L | | 0.32 ± 0.13 | 0.47 ± 0.19 | 0.512 |
|  | 6.Parietal Inf+Supramarginal R | | 0.12 ± 0.10 | 0.30 ± 0.14 | 0.274 |
|  | 7.Fusiform+Occipital Inf L | | 0.39 ± 0.13 | 0.31 ± 0.18 | 0.684 |
|  | 8.Fusiform+Occipital Inf R | | 0.22 ± 0.10 | 0.17 ± 0.15 | 0.776 |
|  | *9.Default Ant (mPFC) bilateral* | | -0.18 ± 0.06 | -0.15 ± 0.09 | 0.764 |
|  | *10.Precuneus+PCC bilateral* | | -0.10 ± 0.05 | -0.07 ± 0.08 | 0.755 |
|  | *11.Angular L* | | -0.48 ± 0.14 | -0.27 ± 0.21 | 0.372 |
|  | *12.Angular R* | | -0.26 ± 0.10 | -0.10 ± 0.14 | 0.321 |
|  | *13.Hippocampus/parahippocampus L* | | -0.11 ± 0.05 | -0.09 ± 0.07 | 0.811 |
|  | *14.Hippocampus/parahippocampus R* | | -0.11 ± 0.06 | -0.18 ± 0.09 | 0.531 |
|  | 15.Precuneus bilateral | | 0.25 ± 0.08 | 0.19 ± 0.11 | 0.595 |
|  | 16.Parietal Sup L | | 0.70 ± 0.19 | 0.61 ± 0.27 | 0.762 |
|  | 17.Parietal Sup R | | 0.48 ± 0.15 | 0.44 ± 0.22 | 0.873 |
|  | 18.Frontal Sup L | | 0.10 ± 0.10 | 0.22 ± 0.14 | 0.458 |
|  | 19.Frontal Sup R | | 0.11 ± 0.09 | 0.21 ± 0.14 | 0.523 |
|  | 20.Frontal Sup Medial bilateral | | 0.03 ± 0.09 | 0.18 ± 0.12 | 0.285 |
| **VCA:**  **Square completion/ Motor contrast** | 1.Frontal Inf L | | 0.74 ± 0.14 | 0.43 ± 0.21 | 0.192 |
|  | 2.Frontal Inf R | | 0.98 ± 0.21 | 0.59 ± 0.31 | 0.272 |
|  | 3.Frontal Mid L | | 0.78 ± 0.14 | 0.47 ± 0.20 | 0.177 |
|  | 4.Frontal Mid R | | 0.99 ± 0.20 | 0.41 ± 0.30 | 0.094 |
|  | 5.Parietal Inf +Supramarginal L | | 1.61 ± 0.23 | 1.19 ± 0.33 | 0.270 |
|  | 6.Parietal Inf+Supramarginal R | | 1.07 ± 0.19 | 0.87 ± 0.28 | 0.532 |
|  | **7.Fusiform+Occipital Inf L** | | **1.95 ± 0.24** | **1.06 ± 0.35** | **0.033** |
|  | **8.Fusiform+Occipital Inf R** | | **1.54 ± 0.22** | **0.77 ± 0.33** | **0.048** |
|  | *9.Default Ant (mPFC) bilateral* | | -0.36 ± 0.11 | -0.48 ± 0.16 | 0.525 |
|  | *10.Precuneus+PCC bilateral* | | -0.51 ± 0.14 | -0.59 ± 0.20 | 0.721 |
|  | *11.Angular L* | | -0.95 ± 0.29 | -0.91 ± 0.43 | 0.930 |
|  | *12.Angular R* | | -0.68 ± 0.19 | -0.69 ± 0.28 | 0.972 |
|  | *13.Hippocampus/parahippocampus L* | | -0.13 ± 0.04 | -0.12 ± 0.06 | 0.799 |
|  | *14.Hippocampus/parahippocampus R* | | -0.10 ± 0.03 | -0.20 ± 0.05 | 0.081 |
|  | **15.Precuneus bilateral** | | **0.85 ± 0.14** | **0.34 ± 0.20** | **0.036** |
|  | 16.Parietal Sup L | | 2.43 ± 0.30 | 1.56 ± 0.43 | 0.090 |
|  | 17.Parietal Sup R | | 1.94 ± 0.33 | 1.61 ± 0.48 | 0.546 |
|  | 18.Frontal Sup L | | 0.53 ± 0.10 | 0.28 ± 0.14 | 0.118 |
|  | **19.Frontal Sup R** | | **0.54 ± 0.09** | **0.21 ± 0.13** | **0.033** |
|  | 20.Frontal Sup Medial bilateral | | 0.30 ± 0.08 | 0.17 ± 0.12 | 0.337 |

**ROI activation defined as (sum of T scores within the ROI among voxels with T scores > 1.66 that are part of clusters of at least 10 voxels) / total voxels in the ROI.

ROI in italics are hypothesized to deactivate under the task. Otherwise, the ROI is hypothesized to activate.

ROI in bold type are significant at p < 0.05.

Mid = Middle, Inf = Inferior, Sup = Superior, R = right, L = left, ACC = anterior cingulate, PCC = posterior cingulate, SMA = supplementary motor area, mPFC = medial prefrontal cortex
